# A Survey on Hand Pose Estimation with Wearable Sensors and Computer-Vision-Based Methods

**DOI:** 10.3390/s20041074

**Published:** 2020-02-16

**Authors:** Weiya Chen, Chenchen Yu, Chenyu Tu, Zehua Lyu, Jing Tang, Shiqi Ou, Yan Fu, Zhidong Xue

**Affiliations:** 1 School of Software Engineering, Huazhong University of Science and Technology, Wuhan 430074, China; chenweiya@isyslab.org (W.C.); cherry_yu@hust.edu.cn (C.Y.); chenyu_tu@hust.edu.cn (C.T.); lvzehua@hust.edu.cn (Z.L.); 2 Ezhou Institute of Engineering, Huazhong University of Science and Technology, Ezhou 436000, China; Tangjing@isyslab.org; 3 School of Mechanical Science and Technology, Huazhong University of Science and Technology, Wuhan 430074, China

**Keywords:** human–computer interaction, computer vision, data gloves, hand pose estimation, deep learning, wearable devices

## Abstract

Real-time sensing and modeling of the human body, especially the hands, is an important research endeavor for various applicative purposes such as in natural human computer interactions. Hand pose estimation is a big academic and technical challenge due to the complex structure and dexterous movement of human hands. Boosted by advancements from both hardware and artificial intelligence, various prototypes of data gloves and computer-vision-based methods have been proposed for accurate and rapid hand pose estimation in recent years. However, existing reviews either focused on data gloves or on vision methods or were even based on a particular type of camera, such as the depth camera. The purpose of this survey is to conduct a comprehensive and timely review of recent research advances in sensor-based hand pose estimation, including wearable and vision-based solutions. Hand kinematic models are firstly discussed. An in-depth review is conducted on data gloves and vision-based sensor systems with corresponding modeling methods. Particularly, this review also discusses deep-learning-based methods, which are very promising in hand pose estimation. Moreover, the advantages and drawbacks of the current hand gesture estimation methods, the applicative scope, and related challenges are also discussed.

## 1. Introduction

With the rapid growth of computer science and related fields, the way that humans interact with computers has evolved towards a more natural and ubiquitous form. Various technologies have been developed to capture users’ facial expressions as well as body movements and postures to serve two types of applications: information captured becomes a “snapshot” of a user for computers to better understand users’ intentions or emotional states; and users apply natural movements instead of using dedicated input devices to send commands for system control or to interact with digital content in a virtual environment.

Among all body parts, we depend heavily on our hands to manipulate objects and communicate with other people in daily life, since hands are dexterous and effective tools with highly developed sensory and motor structures. Therefore, the hand is a critical component for natural human–computer interactions, and many efforts have been made to integrate our hands in the interaction loop for more convenient and comfortable interactive experiences, especially in a multimodal context as demonstrated in the “put-that-there” demonstration [1].

We can use hands for human–computer interaction either directly or through predefined gestures. These two modes have formed two different but highly related issues for hand-based interactions: hand gesture recognition and hand pose estimation. They are both challenging problems to be solved with existing sensing technology because the hand has a high degree of freedom with articulated joints, and because hands can have delicate and rapid movements. Hand gesture recognition is a pattern recognition problem that maps the hand’s appearance and/or motion related features to a gesture vocabulary set, whereas hand pose estimation can be considered as a regression problem that aims to recover the full kinematic structure of hands in 3D space.

Driven by applications like sign language interpretation and gesture-based system control, hand gesture recognition has been extensively studied from early on and there exist many comprehensive reviews [2,3,4,5,6]. Hand gestures, either static or dynamic, can now be successfully recognized if the gesture categories are well defined with proper inter-class distances. Many consumer-level applications, such as the gesture control on Microsoft Hololens [7], can already provide robust recognition performance. Nevertheless, despite sharing some common points with gesture recognition, accurate hand pose estimation of all hand joint, remains a challenging problem.

With the emergence of low-cost depth sensors such as Microsoft Kinect [8] and Intel RealSense [9], and also the boost of machine learning methods, especially the rapid development of convolutional neural networks, there has been considerable progress in hand pose estimation, and state-of-the-art methods can now achieve good performance in a controlled environment. However, hand posture estimation has had much less attention in the literature compared to the recognition. The goal of this paper is to provide a timely overview of the progress in the field of hand pose estimation, including devices and methods proposed in the last few years.

Hand pose estimation can be roughly put into two categories based on the corresponding sensing hardware: wearable sensors and vision-based sensors. While glove-shaped wearable sensors are mostly self-contained and portable, vision-based sensors are very popular since they are more affordable and allow unconstrained finger movements. Both types of devices find their usage under certain circumstances and are still in constant development.

The main contributions of this paper are summarized as follows:

1. Existing surveys focus either on glove-based devices [10,11] or vision-based [12,13,14] systems, since these works were carried out in two distinct research communities, i.e., human–computer interaction and computer vision. We covered both directions to provide a complete overview of the state-of-the-art for hand pose estimation, which can be particularly helpful for people making applications with hand pose estimation technology.
2. With the boost of data-driven machine learning methods, a large number of new solutions have been proposed recently, especially in the last three years. It is now urgent to provide a comprehensive review of current progress to help researchers that are interested in this field to obtain a quick overview of existing solutions and unsolved challenges.

The reminder of the paper is as follows: Section 2 summaries the structural properties of the hand and some intrinsic and extrinsic difficulties related to the pose estimation problem. Section 3 presents different types of glove-shaped wearable sensors to capture finger-level poses, and Section 4 lists hand pose estimation methods and datasets working with various type of cameras. Finally, Section 5 summarizes challenges and possible working directions on this topic.

## 2. Problem Formulation

### 2.1. Hand Structure

The hand is a highly complex and articulated body part, which makes it difficult to model its kinematic and dynamic properties, especially in real time. Thus, here we start from its anatomical structure to arrive at a kinematic model, which is a basic representation of the hand skeleton without consideration of deformation of soft tissues. The kinematic model itself is the basis for the hand pose estimation problem.

**Figure 1:**
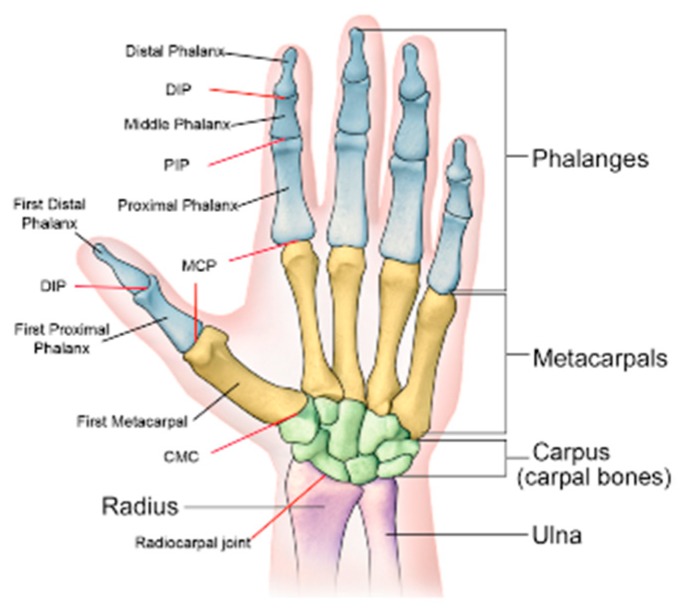
The hand skeleton seen from the palmar side. Originally published in [17]. DIP: distal interphalangeal; PIP: proximal interphalangeal; MCP: Metacarpophalangeal; CMC: Carpometacarpal.

**Figure 2:**
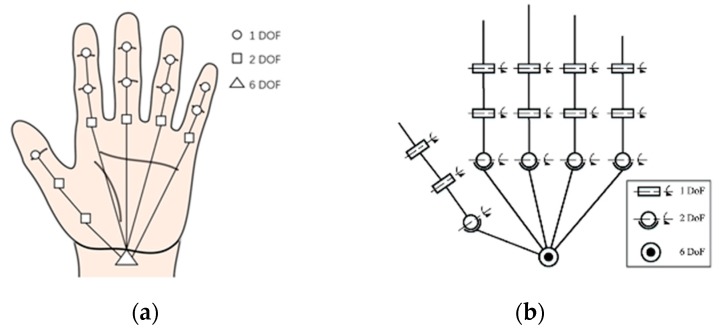
Common kinematic model applied for pose estimation. (**a**) A kinematic hand model with 27 degrees of freedom (DoF) [15]. (**b**) Another kinematic model with 26 DoF [14].

The hand anatomy was introduced to the computer animation community in the 1990s [15]; the hand skeleton seen from the palmar side (named as “Pernkopf Anatomy” [16]) is widely used as a standard to generate kinematic models. The human hand consists of 27 bones belonging to one of the three parts: the wrist, the palm, and fingers, as shown in Figure 1. The bones in the skeleton form a rigid body system with joints having one or more degrees of freedom (DoF) for rotation. The joints between the bones are named as follows from the wrist to the finger tips:

- Carpometacarpal (CMC): joints connecting the metacarpal bones to the wrist;
- Metacarpophalangeal (MCP): joints between the fingers and the palm;
- Interphalangeal (IP): joints between finger segments. They can be further distinguished as distal interphalangeal (DIP) and proximal interphalangeal (PIP);

Getting a clear picture of the hand anatomy can help us to better represent a hand’s configuration in space. A kinematic hand model can be built according to the hand anatomy to encode the hand’s kinematic properties. The IP joints have only flexion–extension ability (1 DoF), and all CMC joints can be considered static (although the CMC of the little and the ring finger have some motion capability reflecting palm folding). However, the thumb is more difficult to model as there exist different considerations regarding the MCP of the thumb (also called the trapeziometacarpal or TM): it can be considered as a 2 DoF saddle joint, as are the other MCP joints that support both abduction/adduction and flexion/extension or having only flexion–extension ability (Figure 2). This leads to two different but very similar kinematic models either with 27 or 26 DoF. Early work from the field of computer animation started by using the 27 DoF model [15], but recent studies all chose the 26 DoF version, which has a simpler modeling of the thumb [18,19,20].

Based on the degrees of freedom analysis, we can use the kinematic model to generate a feature vector to represent a hand’s configuration. More precisely, the 6 DoF frame of the joint connecting the wrist and the hand is often called the global configuration, and the angular DoF of all fingers are called local configurations, which can be combined to form a feature vector for full DoF hand pose estimation.

A kinematic model based on an accurate anatomical structure is a useful way to parameterize the hand, but it is not the only choice. In fact, building a high-resolution anatomical model can be overly complicated for many applications, so various simplifications are proposed in order to keep models only as complicated as needed. For example, the palm can sometimes be represented as a single rigid body if only fingers are of interest [21], although a rigid palm is poor for tasks such as manipulation and grasping. For those tasks, two to four additional DoF can be added for better palm representation [22]. Besides the articulated rigid model, hands can also be modeled as a small group of independent rigid bodies for each component of the hand, and a prior model of the belief propagation network can be used instead to enforce the kinematic relations between these rigid bodies [23].

The kinematic model combined with a shape model are the basis of many model-driven approaches, but the hand can also be modeled in a “non-parametric” way, i.e., an implicit structural model of the hand can be trained from images or other types of data. Different model-based or data-driven methods will be fully discussed in the following sections.

### 2.2. Sensor Taxonomy

The majority of hand pose reconstruction methods are based on either external sensing devices or wearable sensors directly attached to the hand. Although limited in precision, the application of both types of sensors appeared very early in various fields such as gaming, virtual reality, and the related applications, and are still in rapid development.

Wearable sensors are mostly in the form of gloves (also called “data gloves”) that a user can directly put on. Data gloves make use of dedicated electromagnetic or mechanical sensors to directly capture the bending angles of the palm and each finger joint so the local configurations with respect to the wrist can be recorded in real time. As data gloves do not support positional tracking, the global configuration of a hand is often captured with the help of vision-based sensors. 

Vision-based sensors, or more commonly named, cameras, have unprecedented popularity in our daily lives. They can be found on smartphones, drones, humanoid robots, or in the streets and supermarkets, etc. Cameras are ubiquitous tools of low cost to capture a wide range of reflections of visible light, infrared rays, and sometimes lasers. As opposed to wearable sensors, cameras employ indirect measurements by capturing the appearance of the hand from images (pixel arrays) and derive positions of hand joints with intricate algorithms. Recently, with the widespread use of depth cameras (RGB-camera with a depth sensor) and deep learning algorithms, there has been a boost of vision-based methods for hand pose estimation, which in particular leads to this review.

Wearable sensors and vision-based sensors both have some advantages and drawbacks. Vision-based sensors generally do not require the users to wear any devices that may hinder free hand motion; this is particularly important in some real-world applications, such as rehabilitation, a delicate tool manipulation. However, vision-based sensors need the hands to be always visible to the camera and are sensitive to background noise; wearable devices like data gloves are mostly self-contained and mobility-restricted. Thus, these two types of sensors are complementary to each other in hand pose estimation, and more generally in intelligent human–computer interactions. In the following sections, we discuss in detail state-of-the-art methods and commercial solutions of wearable and vision-based sensors for hand pose estimation.

## 3. Wearable Devices

The efforts to develop wearable devices for hand gesture recognition and pose estimation began in the 1970s, and the field has remained active for more than 40 years. This section is mainly focused on late advancements in the two categories of wearable devices for hand pose estimation, namely data gloves and wearable markers. The wearable devices have been reviewed by other surveys [10,24], but gloves designed merely for gesture recognition were not included.

A data glove is a glove-based system composed of one or multiple sensors for data acquisition, and sometimes processing and power supply integration, to be worn on the user’s hands. The bending angle and level of adduction of each finger are captured by embedded sensors of different natures. As summarized by Rashid and Hasan [11], there are typically four types of sensors that can be used for hand-related tasks: bend sensors, stretch sensors, inertial measurement units (IMUs), and magnetic sensors. Most existing data gloves used for hand pose modeling are based on bend or stretch sensors, although some have a combination of multiple types of sensors.

In this section, we present in detail a typical setup and characteristics of data gloves based on bend and stretch sensors as well as other types of sensors.

### 3.1. Bend (Flex) Sensors

Bend or flex sensors are passive resistive devices that are commonly used to measure deflection angles, and are the most widely used among all types of sensors used on hand wearables [25]. Bend sensors are thin and available in different sizes, so they can be easily placed on a glove over the knuckles of finger joints. They also have other advantages such as a relatively long-life cycle and low price, and they can stay operational in a wide range of temperatures, which make them a popular choice to measure different joints of the hand.

Bend sensors can be manufactured by coating resistive carbon elements on a flexible thin plastic substrate or by using optic fibers with mounted receivers. For example, the CyberGlove series gloves are built with conductive-ink-based bend sensors and have been on the market for more than 20 years. The latest CyberGlove III [26] has reached a resolution of less than 1 degree and a data rate of up to 120 records/second. The VPL glove (no longer available) and 5DT glove [27] are also classical data gloves that are based on optical flex sensors. 

**Figure 3:**
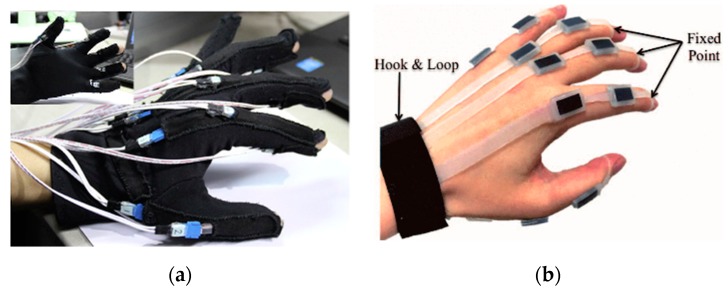
Data gloves based on bend sensors. **(a)** Data glove from Zheng et al. [28]. (**b**) Soft rubber data-collecting glove [29].

Besides commercial products, there are also many research efforts to design gloves based on bend sensors for different applicative purposes. Some glove designs make use of off-the-shelf bend sensors [28] (Figure 3a), whereas others tried to design novel, soft bend sensors [29,30] (Figure 3b). Typical bend sensor-based gloves have up to 22 sensors per hand with reasonable cost and design complexity; a design with a bend sensor array [31] can further increase the number of integrated sensors without hindering natural hand movements.

Bend sensors also have some limitations. Although they can bend millions of times, their accuracy generally decreases over time. Bending a flex sensor with no protective coating for a long period can result in a permanent bend in the sensor, affecting its base resistance. This stability issue requires periodic recalibration, which is not a trivial process. 

### 3.2. Stretch (Strain) Sensors

Stretch sensors are increasingly used for the measurement of human body movements as they can be stretched to fit joints and other deformable parts of the human body and obtain measurements of good quality. With the development of material science and sensing technology, various stretch sensors are proposed in different sizes and sensitivities to fit particular applications, some also with pressure measurement capacity. While non-stretchable data gloves tend to be cumbersome and hinder free hand movements with unsuitable sizes and rigid components, elastic stretch sensors can allow for very slim and comfortable data gloves that fit the hand and are particularly dexterous and sensitive.

Stretch sensors are typically resistors with resistance values directly proportional to the sensor’s deformation. They can be roughly divided into two groups depending on the process of fabrication. They are either made of stretchy fabrics coated with a conducting material such as polymer or metal, or they are constructed by knitting and stitching conductive fiber with resistive thread to form a mixed structure. 

**Figure 4:**
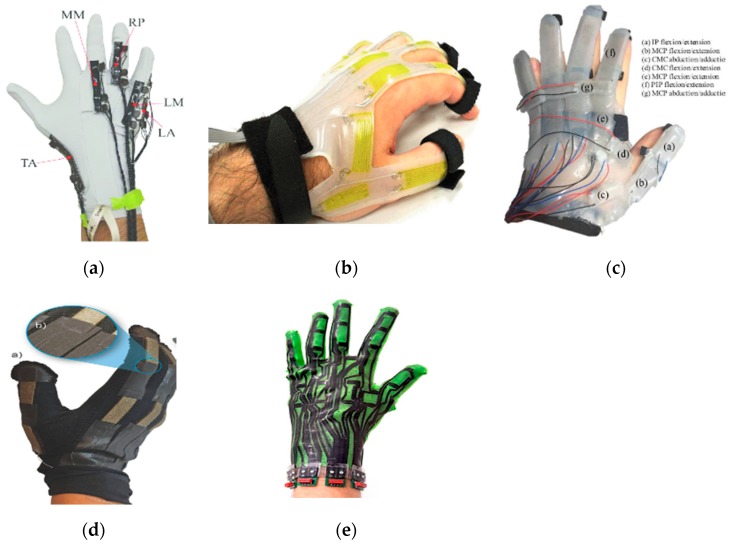
Data glove made with stretch (strain) sensors. (**a**) A kinesthetic glove composed of five piezoresistive fabric (KPF) goniometers [33]; (**b**) wearable soft artificial sensing skin made of a hyperelastic elastomer material [36]; (**c**) data glove made of soft Ecoflex material [37]; (**d**) wearable glove based on highly stretchable textile–silicone capacitive sensors [38]; (**e**) glove made of a full soft composite of a stretchable capacitive silicone sensor array [41]. TA: Thumb Abduction; MM: Middle Metacarpal; RP: Ring Proximal; LM: Little Metacarpal; LA: Little Abduction.

Many recent works have proposed different designs and implementations of stretch sensor gloves. For example, Lee et al. [32] fabricated a stretchable sensor for the detection of tensile as well as compressive strains by putting silver nanoparticle (Ag NP) thin film on a polydimethylsiloxane (PDMS) stamp. Bianchi et al. [33] presented a sensing glove with knitted piezoresistive fabrics (KPFs) (Figure 4a) based on their previous work [34]. This glove is able to track the full hand pose of 19 degrees of freedom (DoF) with only five sensors. Similarly, Michaud et al. [35] built a stretch sensor glove with extremely thin (<50 µm) and skin-conforming sensors made of biphasic, gallium-based metal films embedded in an elastomeric substrate. Besides stretchable fabrics, there are also gloves based on liquid conductors [36,37] (Figure 4b,c) and made with knitted textiles [38,39] (Figure 4d).

However, the abovementioned stretch sensor gloves all have a limited number of embedded sensors (up to 15 [37]), which limits their use for full-hand pose recovery. To solve this problem, Glauser et al. [40] extended the capacitive strain sensor concept of Atalay et al. [38] to achieve dense area-stretch sensor arrays. Later, they designed a stretchable glove based on stretch array sensors, combined with a learned prior, to capture dense surface deformations of full hands [41] (Figure 4e). 

Despite recent advances in stretch sensors, one of their major limitations is that the sensitivity of these sensors changes with the size of the sensor, which makes calibration very difficult. Moreover, stretch sensors exhibit slower response times, and especially, very limited lifespans compared to other sensing technologies.

### 3.3. Other Types of Sensors

Besides bend and stretch sensors, inertial measurement units (IMUs) and magnetic sensing are also very popular.

**Figure 5:**
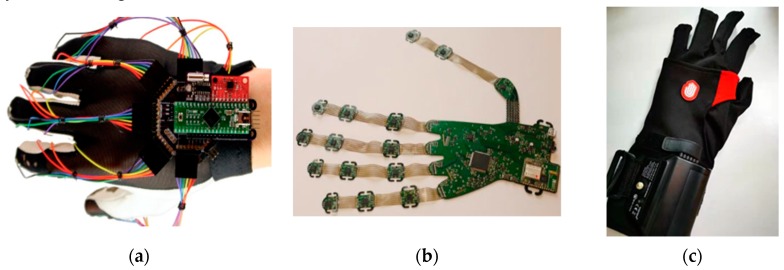
Data gloves made with inertial measurement units (IMUs) or magnetic sensors. (**a**) Keyglove Prototype E [48]. (**b**) IMUs combined with stretchable materials [44]. (**c**) Noitom Hi5 VR glove [49].

IMUs are often a combination of accelerometers, gyroscopes, and sometimes magnetometers to provide measurements of linear accelerations and rotation rates. They are commonly used in wearable devices to obtain the orientation and motion related features of body parts, include hands and fingers [42]. When compared with bend or stretch sensors, IMUs provide good data rates as accelerometers give digital outputs, and they are relatively low cost and have long lifespans. For example, Keyglove (Figure 5a) is an Arduino-powered glove that uses touch combinations to generate keyboard and mouse control codes, which is now an open source kit for further development. Other IMU-based gloves share similar architectures with 17, [43] or 16, 9-axis IMU’s [44] (Figure 5b), where each one includes a 3-axis accelerometer, a 3-axis gyroscope, and a 3-axis magnetometer to provide a real-time measurement of hand joint movements. A recent commercial product named Hi5 VR Glove is designed for VR applications (Figure 5c). It contains 6, 9-axis IMU sensors on each finger for full left-and-right-hand motion capture with high-performance tracking. 

Magnetic sensors, including linear Hall-effect and magnetic current sensors, are also used for hand pose capturing. Due to their contactless working principle, magnetic sensors enable repeatable operations by avoiding frictional forces. Moreover, Hall-effect sensors are of low cost and compact in size and can work in a wide range of temperatures. For example, the Humanglove [45] has 20 Hall-effect sensors that can measure the joint angles of fingers. Wu et al. [46] proposed a wearable rehabilitation robotic hand using Hall-effect sensors that can be worn on the forearm. Another light-weight system called Finexus was designed as a multipoint tracking system by instrumenting the fingertips with electromagnets [47].

Both IMU or magnetic sensors are rigid components and have the problem of tricky sensor placement on the glove as finger tracking requires the sensors to be small enough to be wearable. The sensors have to be placed in between each finger joint to catch poses in detail, which is quite challenging due to their fixed shape and dimensions. Moreover, the sensitivity of magnetic sensors increases with their size, so small sensors are often lacking in precision and are easier to be disturbed by external magnetic fields.

### 3.4. Evaluations

There are seldom direct comparisons between data gloves as most of them are still prototypes in the lab and only a few commercial products exist (some disappeared), so it is difficult to draw conclusions on the reconstruction quality among these solutions.

However, as shown in Table 1, we can still benefit from the analyses of different types of sensors; on one hand, bend (flex) sensors and stretch (strain) sensors are very suitable for hand pose estimation as they are less disturbing for the users with deformable abilities to follow finger movements and palm deformations; on the other hand, IMUs and magnetic sensors have no burdens from mechanical deformation; thus, can have longer lifespans across usage. Thus, the optimal design of a data glove may involve multiple types of sensors to joint their advantages for better performance with lower cost. A further comparison of wearable technologies on accuracy, cost, and lifetime can be found in [11].

## 4. Computer-Vision-Based Methods

The computer vision community has witnessed rapid advancements in almost every sub-domain in recent years, from famous local image descriptors such as SIFT [50], to applicative algorithms like the Adaboost face detection framework [51], and then to the boom of deep learning-based image analyses methods such as Resnet [52] and GAN [53].

The computer-vision-based hand pose estimation has made some progress in recent years. The pose estimation task can be further subdivided into 2D and 3D estimation tasks according to the input data. Deriving 3D hand poses merely from 2D images is extremely difficult due to depth ambiguity and the difficulty of obtaining fully-annotated data for training. The emergence of commodity depth sensors makes pose estimation much easier by solving the depth ambiguity issue, and most recently proposed methods are largely based on depth maps. However, some methods still target pose recovery using merely monocular RGB images, as RGB cameras are widely available since depth sensors bring addition cost and they are limited in the usable range (usually less than 10 m).

Whichever data used, vision-based hand pose estimation methods can generally be grouped into two categories, namely generative and discriminative. Generative methods are also known as model-based or model-driven methods, as they need to construct a 3D hand model based on prior knowledge of the hand structure and are optimized continuously to better fit the shape of the hand. Discriminative methods are also called appearance-based methods or data-driven methods, and they directly predict the joint locations from images to implement hand pose estimation.

The purpose of the hand pose estimation based on model-based methods and the discriminative methods is to obtain a representation of the hand for tracking hand movement. Given a hand image, the main task of the model-based method is to find the optimal parameters of the hand model to fit the hand in the image, and the goal is to model the hand structure in 3D space. The model-based hand pose estimation does not require any datasets to learn the parameters of the hand model. This is different from the discriminative methods, which use a large amount of hand data to train a unified model that can calculate the coordinates of the hand joint points to achieve hand pose estimation. The process of learning and predicting is separated in the discriminative methods, and the way that online learning and offline prediction leads to rapid execution performance. However, in the model-based methods, the parameters of the hand model in each frame need to be re-learned.

In this section, we describe, respectively, common model-based methods and discriminative methods that have been proposed in recent years, as well as the existent problems and improvement methods. The hybrid methods that use both generative and discriminative models are also introduced. At the end of this section, we describe commonly used public datasets for training and benchmark purposes.

### 4.1. Generative Methods

**Figure 6:**
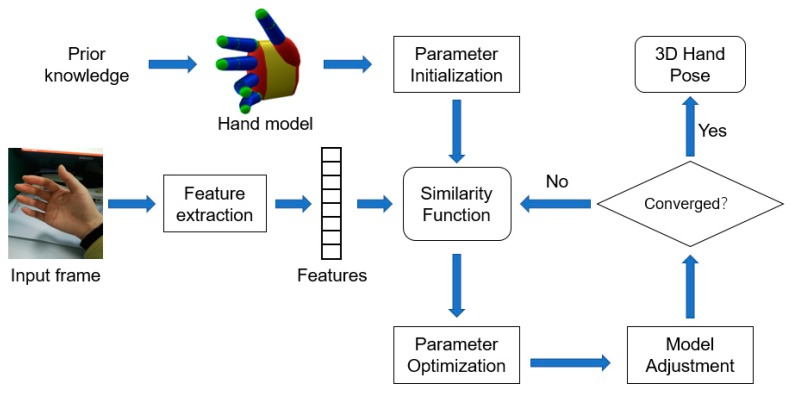
The workflow of generative methods for hand pose estimation.

**Figure 7:**
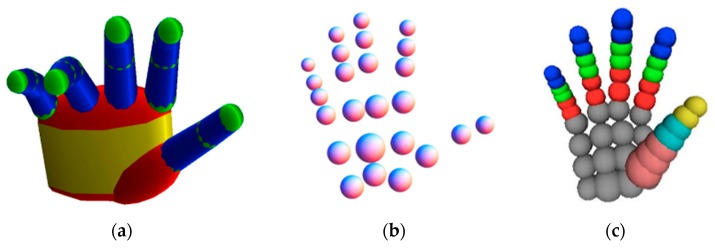
Hand models made of geometric primitives. (**a**) The hand model consisting of color-coded geometric primitives (yellow: elliptic cylinders, red: ellipsoids, green: spheres, blue: cones) [64]. (**b**) The hand’s collision model consisting of 25 spheres [64]. (**c**) Hand model using 48 spheres [19].

**Figure 8:**
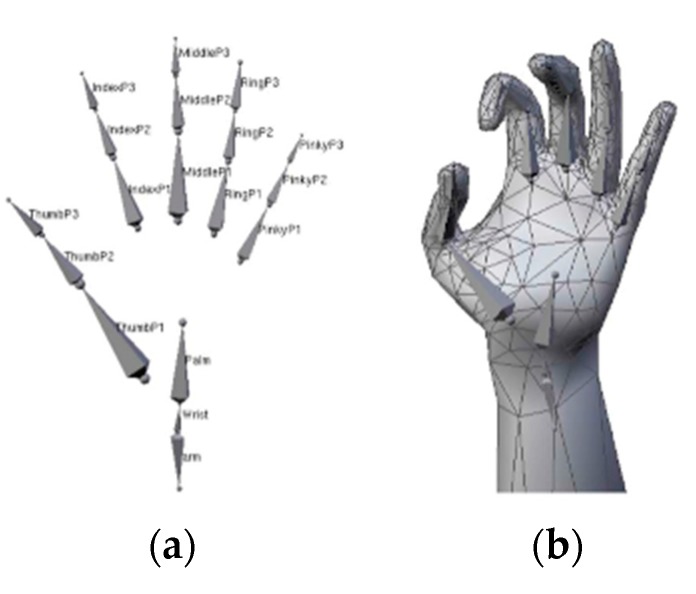
Hand model composed of meshes. (**a**) The skeleton. (**b**) The deformed hand triangulated surface [55].

A generative method needs to construct an explicit hand model based on prior knowledge of the hand structure to recover the hand pose. The hand model needs to satisfy the hand morphology constraints. The task of generative methods is composed of four parts, as shown in Figure 6. Firstly, a hand model should be selected according to the prior knowledge. Different kinds of hand models are shown in Figure 7 and Figure 8. Then, the parameter of the model is to be initialized. The commonly used initialization method is to use the pose from the previous frame as the initialization value of the current frame. After that, a similarity or loss function is established to measure the distance between the actual hand and the chosen hand model, which is represented by hand-crafted features. The commonly used image features are silhouettes, edges, shading, optical flow, and depth value [54,55,56,57,58,59]. At last, parameters of the model are continuously updated until the optimal parameters are found. Commonly used optimization methods are iterative closest point (ICP) [60] and particle swarm optimization (PSO) [61].

The kinematic hand models presented in Section 2.1 are intuitive and accurate hand models for pose fitting tasks, except that their high dimensional nature makes the optimization difficult to solve in real-time; thus, variants of kinematic hand models are often used in discriminative methods rather than model-based methods. Currently, geometric models are often used as the 3D hand model in the generative method-based hand pose estimation. Geometric models are usually composed of some simple geometric primitives such as triangles, cylinders, polygons, or their combination. This way of splitting the hand model into smaller structures largely reduces the dimension of the problem and simplifies the task complexity to a certain extent and is often used in computationally complex model-based hand pose estimation. The most commonly used geometric models are the generalized cylindrical model and the deformable polygonal mesh model.

#### 4.1.1. Generalized Cylindrical Model

Oikonomidis et al., as pioneers, used the generalized cylindrical model (GCM) to achieve generative method-based hand pose estimation [62]. In their work, the hand model they used consisted of four kinds of geometric primitives: cylinders, ellipsoids, spheres, and cones. The hand model is shown in Figure 7a, which has 26 DOF and 27 parameters. It uses the skin and edge feature maps to measure the differences between hand model and the true hand with PSO as the optimization method. The paper points out that it proves for the first time that PSO can be used for hand pose estimation and can achieve certain accuracy and robustness. 

Compared to RGB images, RGB-D images can provide depth information as an additional source to reduce the computational complexity of hand pose estimation and can be more robust to illumination changes. Thus, Oikonomidis et al. [58] proposed to use skin information and depth information from RGB-D image. In the proposed method, first the RGB image and the depth image are obtained from a Kinect. Then, the hand is segmented by combining the skin color information and the depth information. Finally, the hand model is used to fit the real hand by optimization with PSO.

However, this work can track only one hand, so they further proposed a method that can track the full articulation of two hands from a video sequence [63]. The objective function calculates the distance between the image and the hand model based on the image depth value and color and uses a PSO search heuristic to optimize the objective function. The method enables tracking of two interacting hands with an accuracy of 6 mm.

Oikonomidis et al. further proposed a method for estimating hand pose under the conditions of interactions between human hands and objects in the work [64]. In addition to the hand model shown in Figure 7a, a hand collision model consisting of 25 spheres, shown in Figure 7b, was also proposed to keep track of interactions between the hand and the object.

Due to the fast motion of the hand, the initialization method based on the pose of the last frame is not good enough. Qian et al. [19] proposed a method that can first detect the fingers to generate intermediate poses to help hand initialization. In their research, a hand model consisting of 48 simplest spheres was used to estimate the hand pose, as shown in Figure 7c. They pointed out that gradient-based optimization and manual tracking optimization based on random tracking are not good enough to minimize the cost function. They are either too sensitive to local minima or too slow to converge. Observing the complementarity of the two methods, a hybrid local optimization method ICP–PSO was used in the optimization process, converging faster computation and better resisting local optima.

#### 4.1.2. Deformable Polygonal Mesh Model

The deformable polygonal mesh model (DPMM) usually consists of a surface model and an underlying skeleton model. In the parameter calculation process, a specific method is needed to deform the surface model according to pose changes of an underlying articulated skeleton.

In order to recover a 3D hand from only RGB images, de La Gorce et al. [55] proposed a deformed hand triangulated surface, which had 28 DoF and was deformed according to pose changes of an underlying articulated skeleton using skeleton subspace deformation [65,66]. The model is shown in Figure 8. The proposed objective function can handle self-occlusion and illumination problems, and explicitly use temporal texture continuity and shadow information at the same time. It minimizes the objective function using quasi-Newton methods. In each frame, the parameters of the hand model are initialized using the results of the previous frame.

The quasi-Newton method was used in the optimization process for the method mentioned above. This is a local optimization method, which is more efficient but requires accurate design of the objective function to avoid local minima. Ballan et al. [67] proposed a generative approach based on local optimization that uses a discriminatively trained salient point detector to achieve better accuracy. This method adds edges, optical flow, and collision information to the objective function, and can detect the interaction between two hands and objects. The proposed hand model consists of a surface mesh model and an underlying bone skeleton, and the surface deformations are encoded using the linear blend skinning operator (LBS) [66]. In each frame, the positions of the fingernails are detected using the Hough Forest classifier as the salient points. These salient points are used to help find the hand position during the interaction and to make a distinction between two hands. However, the method needs heavy computations and has poor real-time performance. 

Different from the work above, Sridhar et al. [68] proposed a faster method that uses the linear Support Vector Machine (SVM) classifier as the discriminator to find the fingertip position in the depth map. The proposed hand model is the SoG (sum of Gaussian) model, and the color information is used to calculate the hand model parameters; then a gradient descent method is used to optimize the parameters of the hand model. Tzionas et al. [69] also used the linear blend skinning (LBS) [66] model, which consists of a triangular mesh and an underlying kinematic skeleton. The method uses the information from an RGB-D image to track two interacting hands. This method only uses an RGB-D camera to realize hand pose estimation, while the work of Ballan et al. [67] needed a more expensive and elaborate multi-camera system.

In the generative methods, the commonly used data types are an RGB image and an RGB-D image. The commonly used optimization techniques are PSO. The important information of generative methods is summarized in Table 2 and Table 3.

### 4.2. Discriminative Methods

The goal of discriminative methods is to learn a map from visual features to the target parameter space, such as joint labels or joint 3D locations from images or videos. Discriminative methods rely heavily on the quality of training data as they require one or more datasets to train the model; the labels of datasets give the position of the joint of the hand. The goal of model prediction is to compute the coordinates of the hand joints in the image.

There are two major types of discriminative methods: random forests (RF)- and convolutional neural network (CNN)-based models.

#### 4.2.1. Random Forest

Methods based on random forests [70] consider hand pose estimation as a regression problem. This line of work was pioneered by Keskin et al. [71], who used a randomized decision forest (RDF) for hand shape classification and applied this shape classification forest (SCF) to a novel multi-layer RDF framework for hand pose estimation. This classifier assigns the input depth pixels to hand shape classes and directs them to the corresponding hand pose estimators trained specifically for that hand shape.

However, the above approach needs large amounts of per-pixel labeled training data, which is difficult to obtain, so it extensively uses synthetic data in training that leads to performance discrepancies among realistic and synthetic pose data. To tackle this problem, Tang et al. [72] proposed the semi-supervised transductive regression (STR) forest to learn the relationship between a small, sparsely labelled realistic dataset and a large synthetic dataset using transductive learning. They also designed a novel data-driven, pseudo-kinematic technique to refine noisy or occluded joints.

Pixel-level classification is often prone to noisy real world data, so Liang et al. [73] used a superpixel-Markov random field (SMRF) parsing scheme to enforce the spatial smoothness and the label co-occurrence prior to remove the misclassified regions. They targeted the robustness of regression with more discriminative depth-context features by using a novel distance-adaptive selection method.

**Figure 9:**
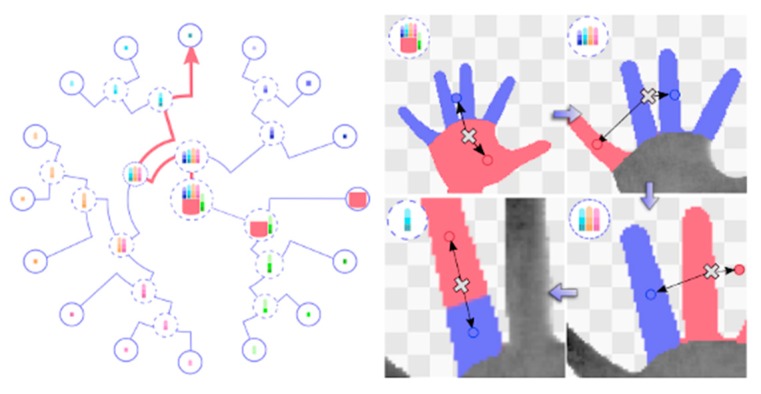
A binary latent tree model (LTM)-based [75] search process for skeletal joint position estimation.

To further improve the accuracy and efficiency of the regression forest-based method, Tang et al. [74] proposed a new forest-based, discriminative framework for structured searches in images called latent regression forest (LRF). The method takes a depth map as input and learns the topology of the hand with unsupervised learning in a data driven manner. The main difference of LRF from existing methods is that it employs a structured coarse-to-fine search on a point cloud instead of dense pixels, and an error regression step to avoid error accumulation. As shown in Figure 9, once LRF is trained, point-region correspondence can be found by a tree search in a divide-and-conquer way.

Instead of performing regression for all hand joints, one may employ a progressive strategy via a sequence of weak regressors [76]. Based on this idea, Sun et al. [77] proposed a cascaded regression method for hand pose estimation. Their key observation is that different object parts typically exhibit different amount of variations and degrees of freedom due to the articulated structure. Thus, regressing all parts together is unnecessarily difficult and causes slow convergence and degraded accuracy. Their hierarchical approach regresses the pose of different parts sequentially in the order of their articulation complexity. Similarly, Wan et al. [78] designed a hierarchical regression framework for estimating hand joint positions from single depth images following the tree structured topology of the hand from wrist to finger tips. They proposed a conditional regression forest, i.e., the frame conditioned regression forest (FCRF) along with local surface normals instead of normal difference as features. This modification was shown to obtain consistent improvement over previous discriminative pose estimation methods on real-world datasets.

#### 4.2.2. Convolution Neural Networks

Deep learning has developed rapidly in recent years and has been widely used for hand pose estimation. This type of method trains deep convolutional neural networks and learns model parameters through a large number of labeled datasets so that it can predict the joint locations to achieve hand pose estimation.

Tompson et al. [79] proposed a four stage method for hand pose estimation. First, the input image was processed by the decision forest to separate the hand from the background. When the hand in the image was acquired, a robust method was developed to label the dataset. After that, a deep convolutional neural network was used to extract the heatmap from the input hand image. Finally, the features were extracted from heatmaps and an objective function was proposed and minimized to align the features of the model to heatmap features. 

**Figure 10:**
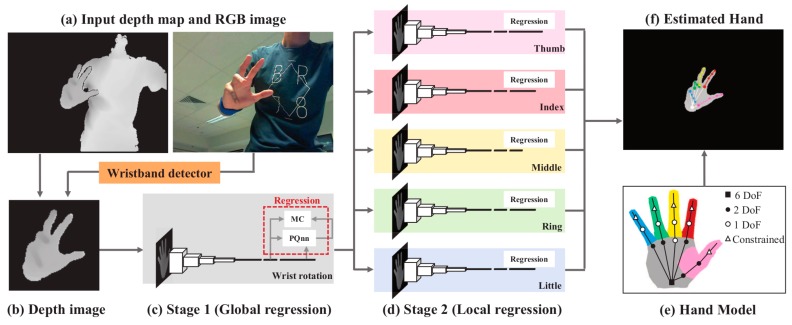
Global regression calculates the wrist parameters, local regression calculates five fingers parameters [20].

Although the method above shows good result in hand tracking, it is inefficient in situations with occlusion, because it uses the inverse kinematic (IK) approach to recover a 3D pose from a 2D image. To solve this problem, Sinha et al. [20] proposed a method based on global and local regression. In their work, parameters of the wrist were computed in global regression, and then the parameters of five fingers were separately calculated using five local regression networks, which is shown in Figure 10. This method can effectively deal with occlusion problems, and it can also avoid the need to re-initialize all parameters when the previous frame is lost.

The work above only considered predicting the positions of hand joints directly. However, during hand movement, there is a strong correlation between different hand joints, so prior information can be introduced to constrain the parameter space. The method proposed by Oberweger et al. [80] adds prior information to predict the parameters of the pose in a lower dimensional space, and can solve the ambiguity of the finger joints. They introduced a “bottleneck” structure to the last layer of the network, which is a layer with only necessary neurons.

Although the works above solve the occlusion problem or use prior information to constrain the parameter space to achieve good results, they are in general very demanding on the training dataset. To reduce the cost of getting large amounts of labelled data from the real world, they often use synthetic data to train the convolutional neural network. For example, Ge et al. [81] used a synthetic dataset containing both ground truth 3D meshes and 3D poses to realize 3D hand shape and pose estimation. Wan et al. [82] used depth maps, which were generated online from a hand model provided by [45] to train the deep neural network. 

**Figure 11:**
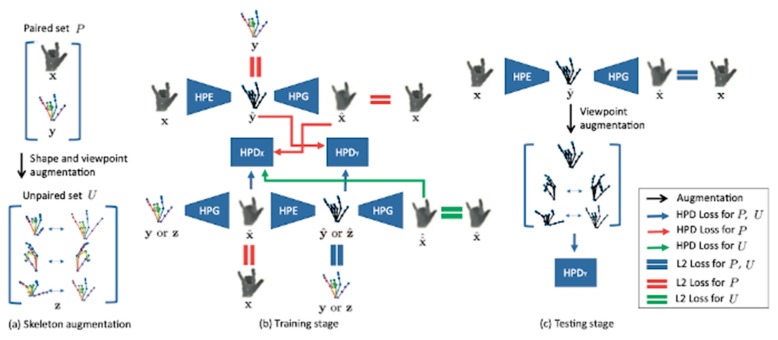
The augmented skeleton space model [83]. HPE: hand pose estimator; HPG: hand pose generator; HPD: hand pose discriminator.

Due to the gap between synthetic and real data, the models trained with synthetic data often have poor performance once applied in the real-world. Although we are aware of the importance of real data, building a dataset covering all possible camera viewpoints and hand poses with detailed annotations is still a big challenge. To build a functional model without a large training dataset, Baek et al. [83] proposed a method for synthesizing data using skeleton maps to add data to the skeleton space. As shown in Figure 11, the model consists of a hand pose estimator (HPE), a hand pose generator (HPG), and a hand pose discriminator (HPD). This method expands the existing dataset and proposes a method of generating depth map data based on the skeleton map. The simultaneous data generation and model training philosophy yields good prediction results. However, this method still imposes some constraints on the dataset that initiates the model. If the input skeleton map differs greatly from the maps in the dataset during the test, the generated depth map will blur, and the final prediction result will be affected. 

Thus, further efforts were taken in this direction. Oberweger et al. [84] proposed a joint hand–object pose estimation approach that learns a synthesizer CNN to synthesize an image in the model. The synthesizer CNN can generate convincing depth images for a very large range of poses. The method introduces a feedback loop to refine the hand pose estimates. Yang and Yao [85] proposed a method to better deal with the problem of large discrepancies between backgrounds and camera viewpoints. The work proposed the use of disentangled representations and a disentangled variational autoencoder (dVAE) that can synthesize highly realistic images. Spurr et al. [86] developed a generative deep neural network to learn a latent space, which can be used directly to estimate 3D hand poses.

The above-mentioned discriminative methods are summarized in Table 4. There are also some works that can track simultaneously the human body, hand, and face. The convolutional pose machine (CPM) [87] trained with datasets such as FLIC [88], LSP [89], and MPII [90], can deal with cases where there are multiple human bodies and hands in the scene. It aims at single and multi-person body pose estimation and can make good predictions for hand joints location. The Perceptual Computing Lab at Carnegie Mellon University proposed a multi-task 2D human pose estimation method named OpenPose [91], which uses a multi-stage approach to estimate poses for human bodies, faces, and hands, where the hand pose estimation is based on the improvements of CPM. As a multi-network approach, it directly uses existing body, face, and hand key point detection algorithms. Based on the OpenPose project, Hidalgo et al. [92] combined multi-task learning (MTL), which is a classic machine learning technique [93,94,95], and the improved OpenPose model was used to train the first single-network for 2D integral estimation. This method combines multiple independent key point detection tasks into a unified framework that simultaneously detects key points of the body like feet, face, and hands. For the part of the hand pose estimation, the dataset used is the OpenPose hand dataset [96], which combines a subset of 1k hand forms manually annotated from MPII [90], as well as the 15k samples automatically annotated on the Dome or Panoptic Studio [97].

Compared with those methods that leverage a depth map coming from commodity depth sensors, as shown in Table 4, obtaining a 3D hand pose from merely RGB images is generally more challenging than pose recovery from RGB and depth information. As Table 5 shows, Zimmermann and Brox pioneered in this direction by proposing a deep network that learns a network-implicit 3D articulation prior [98]. Iqbal et al. proposed a novel 2.5D pose representation and implicitly learned depth map and heatmap distributions with a novel CNN architecture [99]. However, these methods require large amounts of annotated data, which are difficult to generate, and synthetic datasets are used instead. To ensure good generalization ability to real hands, Rad et al. learned a mapping from paired color and depth images and aligned synthetic depth images with the real depth images [100]. Cai et al. used a weakly-supervised method that adapts from a fully-annotated synthetic dataset to a weakly-labeled real-world dataset with the aid of a depth regularizer [101]. Recently, Ge et al. made further improvements by proposing a graph CNN-based method to reconstruct both 3D hand poses and shapes represented by a full 3D mesh [81].

There are also some very recent works that brought new insights into the field by employing CNN with custom modifications. CNNs can be applied to images from multiple viewpoints [102], combined with octrees [103,104], or applied to a point cloud instead of pixels [105,106,107,108], or even with a complete 3D architecture [109,110,111,112].

### 4.3. Hybrid Methods

Generative methods need to re-compute the parameters of the hand model for each frame, the speed of which is slow and thus the real-time performance is usually poor. Moreover, the parameter of each frame of the hand model is often initialized based on the parameters of the previous frame. If the previous frame estimation has an error, this error will accumulate along the running process, thereby affecting the final quality of hand pose estimation.

Although the parameters of the model can be trained offline and used directly in prediction, discriminant methods require a large amount of annotated data to train the model. If the scenes used for training and testing are quite different, the quality of hand pose estimation will also be compromised.

Therefore, some researchers attempted to combine model-based and data-driven approaches. Xu and Cheng [18] used a single depth image and adopted the Hough forest model in a two-stage hand detection method. First the Hough forest model is used to provide an initial estimate of the direction and 3D position of the hand in the plane, then another Hough forest regression model, which is based on the hand coordinates and direction values acquired in the first step, is used to calculate the depth features that are invariant to the rotation in the plane. Next it uses the hand 3D model to generate a reasonable set of 3D candidate gestures. Finally, based on the candidate gesture, the pose estimation is performed by solving the optimization problem. The method uses a skinned mesh model combined with a discriminative approach to achieve hand pose estimation.

Baek et al. [119] proposed a model that is able to estimate the 3D skeleton structure of the hand from the RGB image and recover the hand shape from it. In their work, a 2D skeleton model was used to predict 21 joint points, and the 3D hand model used a generative mesh model named MANO [120] representing the hand grid based on 45-dimensional pose parameters and 10-dimensional shape parameters, which was used in some very recent work [121,122]. The model consists of three parts, namely a 2D evidence estimator to calculate the 2D skeleton coordinates of the hand according to the RGB image, a 3D mesh estimator to compute the 3D mesh model of the hand, and a projector that combines the 3D model information with the hand skeleton coordinate information to obtain the coordinates of 3D hand joints. Another work from Zhang et al. [123] predicted the current hand pose based on the previous poses by a pre-trained LSTM network, which is an interesting way to generate a “hand model” from past experiences.

### 4.4. Public Datasets

At present, most hand pose estimation tasks take place under controlled conditions. Different camera viewpoints, hand poses and shapes, and illuminations and backgrounds are all required to be covered by the training dataset in order to obtain successful hand pose estimation results. However, so far, the variability and quantity in the existing datasets are still relatively limited.

The datasets used in the current literatures include RGB images, depth images (depth maps), and their combination (RGB-D). For different data types, the corresponding labels and annotations in the datasets are also different. The datasets widely used in recent years are summarized in Table 6.

As shown in Table 3 and Table 4, depth data is becoming more and more popular in hand pose estimation tasks as it has good resistance to color and illumination change in the scene and can help extract hands from cluttered backgrounds. Commercial depth sensors such as Kinect and Intel RealSense have relatively good depth sensing performance, although the obtained depth maps are often degraded by noise. We can also see that some datasets contain purely synthetic data and others are constructed with real image data, but manual labeling is not always possible. Thus, to further improve the quality and ability to generalize to unseen situations for discriminative methods, we can continue to pursue hybrid methods that are less dependent on the training datasets, especially how discriminative methods can help hand model initialization and fast calibration. Another direction we can take is to develop weakly-supervised methods that are less demanding for large amounts of labeled training data.

## 5. Challenges and Future Work

From the analyses above, we can see that existing hand pose estimation systems can already accurately track the movement of the human hand in real time in a relatively controllable environment. However, hand pose estimation cannot yet be considered as a solved problem and still faces many challenges, especially in open and complex environments, where we should take the amount of computing resources needed into consideration.

### 5.1. Challenges

Wearable sensors, or data gloves, are promising for accurate and disturbance-free hand modeling since they generally have compact design and become lighter and less cumbersome for dexterous hand movements. However, there are three main challenges remaining to be solved.

Most data gloves are still “in the lab” and there is no industrial standard on the design and fabrication of such devices, which leads to high costs of available commercial products, making them unaffordable for daily use. Second, except gloves that are based on stretch sensors, most gloves have fixed size and are difficult to match different users’ hands. Lastly, gloves are unsuitable to be used in certain cases, for example, some stroke patients have difficulties opening their hands to wear gloves designed for normal users, or in situations when the user needs to manipulate tiny objects, or put their hands into water, etc.

Vision-based methods, on the other hand, have overcome many difficulties faced by common computer vision tasks, such as rotation, scale and illumination invariance, and cluttered backgrounds. The high dimensional nature of hand pose representation, and even hand self-occlusion, are no longer obstacles in the way of achieving accurate hand pose estimation in real time. However, vision-based methods still face the following challenges:

First, occlusion is still the major problem. As the hands are extensively used to manipulate objects in daily life, they are very likely to be blocked or partially blocked by objects during interaction, which forms the hand–object–interaction (HOI) problem. There are already some efforts to deal with object occlusion. For example, Tekin et al. [127] proposed an end-to-end architecture to jointly estimates the 3D hand and object poses from egocentric RGB images. Myanganbayar et al. [128] proposed a challenging dataset consisting of hands interacting with 148 objects as a novel benchmark for HOI.

Second, since many methods are data-driven, the quality and coverage of training datasets is of great importance. As discussed in Section 4.4, there are already many useful datasets with 2D/3D annotations. However, a larger portion of annotated data comes from synthetic simulations. Existing methods tried to employ weakly supervised learning, transfer learning, or different data augmentation approaches to better cope with insufficiency of real world data, but more data representing tremendous viewpoints, shapes, illumination, background variations, and objects in interaction are required to train deep learning-based architecture, or we must find a new way to incorporate the hand model for 3D pose recovery.

Moreover, most deep learning-based methods also require large amounts of computational resources during the training and inference stages. Many algorithms need to run on a graphics processing unit (GPU) to achieve a real-time frame rate, making it difficult to be deployed to portable devices such as mobile phones and tablets. Thus, it is important to find effective and efficient solutions on mobile platforms for ubiquitous applications.

### 5.2. Future Work

To conclude, various devices and methods have already enabled hand pose estimation for different applicative purposes in a controlled environment, and we are not far from real-time, efficient, and ubiquitous hand modeling.

In the near future, expertise from material science and electronics is needed to build easy to wear and maintain, yet more affordable data gloves for accurate hand modeling. Regarding vision-based methods, data-efficient methods such as weakly supervised learning or hybrid methods are needed to minimize the dependency on large hand pose datasets and to improve the generalization ability to unseen situations. Moreover, we can already see the benefits of new sensors, e.g., the depth sensor, as they can largely reduce the computation complexity by using 2D data to deduce 3D poses; thus, novel accurate long-range 3D sensors will definitely contribute to contactless hand pose estimation. 

## Figures and Tables

**Table 1:** Comparison between different types of wearable sensors.

Type	Accuracy	Response time	Lifetime	Cost	Ease of Wearing
Bend (Flex) sensor	high	medium	medium	medium	medium
Stretch (Strain) sensor	medium	slow	short	low	easy
IMU	medium	fast	long	low	hard
Magnetic sensor	low	fast	long	medium	hard

**Table 2:** Summary of generative methods for hand pose estimation with RGB input.

Literature	Features	Hand Model	DoF	Parameters	Optimization Method	FPS
Oikonomidis et al. [62]	Skin and edge	GCM ^1^	26	27	PSO	-
Oikonomidis et al. [64]	Skin and edge	GCM	26	27	PSO	-
Gorce et al. [55]	Surface texture and illuminant	DPMM ^2^	22	-	Quasi-Newton method	40
Ballan et al. [67]	Skin, edges, optical flow, and collisions	DPMM	35	-	Levenberg–Marquard	50

^1^ GCM: Generalized Cylindrical Model. ^2^ DPMM: Deformable Polygonal Mesh Model.

**Table 3:** Summary of generative methods for hand pose estimation with RGB and depth inputs.

Literature	Features	Hand Model	DoF	Parameters	Optimization Method	FPS
Oikonomidis et al. [58]	Skin and depth	GCM	26	27	PSO	15
Oikonomidis et al. [63]	Skin and depth	GCM	26	27	PSO	4
Qian et al. [19]	Depth	GCM	26	26	ICP–PSO	25
Sridhar et al. [68]	Skin and depth	DPMM	26	-	Gradient ascent	10
Tzionas et al. [69]	Skin and depth	DPMM	37	-	Self-build method	60

**Table 4:** Summary of discriminative methods for hand pose estimation with RGB and depth inputs.

Literature	Datasets	Method	FPS
Keskin et al. [71]	Self-built dataset	RF: Random decision	-
Tang et al. [72]	Self-built dataset	RF: STR	25
Liang et al. [73]	Self-built dataset	RF: SMRF	-
Tang et al. [74]	Self-built dataset	RF: LRF	62.5
Sun et al. [77]	Self-built dataset	RF: Cascaded regression	300
Wan et al. [78]	-	RF: FCRF	29.4
Tompson et al. [79]	Self-built dataset	RDF + CNN	24.9
Sinha et al. [20]	Dexter1 [68], NYU	CNN: DeepHand	32
Oberweger et al. [80]	NYU, ICVL	CNN: Deep-Prior	500
Ge et al. [102]	MSRA, NYU	CNN: Multi-View CNNs	82
Che et al. [104]	NYU, ICVL	CNN: HHLN and WR-OCNN	-
Ge et al. [105]	NYU, ICVL, MSRA	CNN	41.8
Ge et al. [106]	NYU, ICVL, MSRA	CNN	48
Dou et al. [107]	NYU, MSRA	CNN	70
Li and Lee [108]	NYU, Hands 2017Challenge dataset [113]	CNN	-
Deng et al. [109]	NYU, ICVL	CNN: Hand3d	30
Ge et al. [110]	MSRA, NYU	CNN	215
Moon et al. [111]	ICVL, MSRA, NYU, HANDS2017 [113], ITOP [114]	CNN: 3D CNN	35
Ge et al. [112]	MSRA, NYU, ICVL	CNN: 3D CNN	91

**Table 5:** Summary of discriminative methods for hand pose estimation with RGB input.

Literature	Datasets	Method	FPS
Zimmermann and Brox [98]	Stereo hand pose (STB) [115], Dexter [116], Rendered hand (RHD) [98]	CNN: HandSegNet, PoseNet	-
Iqbal et al. [99]	Dexter [116], EgoDexter [117], STB, RHD, MPII + NZSL [96]	CNN	150
Rad et al. [100]	LINEMOD [118], STB, RHD	CNN, FCN	116
Cai et al. [101]	STB, RHD	CNN	-
Ge et al. [81]	STB, RHD	Graph CNN	50

**Table 6:** Commonly used public datasets for vision-based hand pose estimation.

Dataset	Image Type	Number of Images	Camera	Number of Annotated Joints	Description
ICVL [74]	D	331,000	Intel Creative Gesture Camera	16	Real hand and manual labeling
NYU [79]	RGB-D	81,009	Prime Sense Carmine 1.09	36	Real hand and automatic labeling
BigHand 2.2M [124]	D	2.2M	Intel RealSense SR300	21	Real hand and automatic labeling
HandNet [125]	D	12,773	Intel RealSense Camera	Fingertip and palm coordinates	Real hand and automatic labeling
MSRC [126]	D	10,2000	-	22	Synthetic data
MSHD [126]	D	101k	Kinect2	-	Synthetic data
MSRA14 [19]	D	2400	-	21	Real hand and manual labeling
MSRA15 [77]	D	76,500	Intel’s Creative Interactive Camera	21	Real hand and semi-automatic labeling
OpenPose hand dataset [96]	RGB	16k	-	21	Manual labeling from MPII [90] and automatic labeling on the Dome or Panoptic Studio [97]
Stereo hand pose (STB) [115]	RGB	18,000 frame pairs	Point Grey Bumblebee2 Stereo Camera	21	Real-world stereo image pairs with two subsets: STB–BB and STB–SK
Rendered hand (RHD) [98]	RGB-D	43,986	-	21	Synthetic dataset with 20 different characters performing 39 actions in different settings

## References

[1] Bolt R.A. (1980). “Put-That-There”: Voice and Gesture at the Graphics Interface. SIGGRAPH Comput. Graph..

[2] Rautaray S.S., Agrawal A. (2015). Vision based hand gesture recognition for human computer interaction: A survey. Artif. Intell. Rev..

[3] Zhao X., Zhao J., Fan B., Hao L. Survey on Hand Gesture Recognition and its Application Prospect. Proceedings of the 11th National Conference on Signal and Intelligent Information Processing and Application.

[4] Al-Shamayleh A.S., Ahmad R., Abushariah M.A., Alam K.A., Jomhari N. (2018). A systematic literature review on vision based gesture recognition techniques. Multimed. Tools Appl..

[5] Cheok M.J., Omar Z., Jaward M.H. (2019). A review of hand gesture and sign language recognition techniques. Int. J. Mach. Learn. Cybern..

[6] Park J., Jin Y., Cho S., Sung Y., Cho K. (2019). Advanced machine learning for gesture learning and recognition based on intelligent big data of heterogeneous sensors. Symmetry.

[7] Hololens 2 From Microsoft. https://www.microsoft.com/en-us/hololens/.

[8] Kinect V2, Microsoft. http://www.k4w.cn/.

[9] Realsense Cameras, Intel. https://www.intel.com/content/www/us/en/architecture-and-technology/realsense-overview.html.

[10] Dipietro L., Sabatini A.M., Dario P. (2008). A survey of glove-based systems and their applications. IEEE Trans. Syst. Man Cybern. Part C Appl. Rev..

[11] Rashid A., Hasan O. (2019). Wearable technologies for hand joints monitoring for rehabilitation: A survey. Microelectron. J..

[12] Erol A., Bebis G., Nicolescu M., Boyle R.D., Twombly X. (2007). Vision-based hand pose estimation: A review. Comput. Vis. Image Underst..

[13] Supancic J.S., Rogez G., Yang Y., Shotton J., Ramanan D. Depth-Based Hand Pose Estimation: Data, Methods, and Challenges. Proceedings of the IEEE International Conference on Computer Vision.

[14] Li R., Liu Z., Tan J. (2019). A survey on 3D hand pose estimation: Cameras, methods, and datasets. Pattern Recognit..

[15] Lee J., Kunii T.L. (1993). Constraint-based hand animation. Models and Techniques in Computer Animation.

[16] Pernkopf E. (1989). Pernkopf Anatomy: Thorax, Abdomen, and Extremities.

[17] Wheatland N., Wang Y., Song H., Neff M., Zordan V., Jörg S. (2015). State of the art in hand and finger modeling and animation. Computer Graphics Forum.

[18] Xu C., Cheng L. Efficient Hand Pose Estimation from a Single Depth Image. Proceedings of the IEEE International Conference on Computer Vision.

[19] Qian C., Sun X., Wei Y., Tang X., Sun J. Realtime and Robust Hand Tracking from Depth. Proceedings of the IEEE Conference on Computer Vision and Pattern Recognition.

[20] Sinha A., Choi C., Ramani K. Deephand: Robust Hand Pose Estimation by Completing a Matrix Imputed with Deep Features. Proceedings of the IEEE Conference on Computer Vision and Pattern Recognition.

[21] McDonald J., Toro J., Alkoby K., Berthiaume A., Carter R., Chomwong P., Christopher J., Davidson M.J., Furst J., Konie B. (2001). An improved articulated model of the human hand. Vis. Comput..

[22] Andrews S., Kry P.G. (2013). Goal directed multi-finger manipulation: Control policies and analysis. Comput. Graph..

[23] Sudderth E.B., Mandel M.I., Freeman W.T., Willsky A.S. Visual Hand Tracking using Nonparametric Belief Propagation. Proceedings of the 2004 Conference on Computer Vision and Pattern Recognition Workshop.

[24] Sturman D.J., Zeltzer D. (1994). A survey of glove-based input. IEEE Comput. Graph. Appl..

[25] Saggio G., Riillo F., Sbernini L., Quitadamo L.R. (2015). Resistive flex sensors: A survey. Smart Mater. Struct..

[26] Cyberglove III, CyberGlove Systems. http://www.cyberglovesystems.com/cyberglove-iii.

[27] 5DT Data Glove Ultra Series, 5DT Inc.. http://www.5dt.com/downloads/dataglove/ultra/5DTDataGloveUltraDatasheet.pdf.

[28] Zheng Y., Peng Y., Wang G., Liu X., Dong X., Wang J. (2016). Development and evaluation of a sensor glove for hand function assessment and preliminary attempts at assessing hand coordination. J. Meas..

[29] Shen Z., Yi J., Li X., Lo M.H.P., Chen M.Z., Hu Y., Wang Z. (2016). A soft stretchable bending sensor and data glove applications. Robot. Biomim..

[30] Ciotti S., Battaglia E., Carbonaro N., Bicchi A., Tognetti A., Bianchi M. (2016). A synergy-based optimally designed sensing glove for functional grasp recognition. Sensors.

[31] Saggio G. (2014). A novel array of flex sensors for a goniometric glove. Sens. Actuators A Phys..

[32] Lee J., Kim S., Lee J., Yang D., Park B.C., Ryu S., Park I. (2014). A stretchable strain sensor based on a metal nanoparticle thin film for human motion detection. Nanoscale.

[33] Bianchi M., Haschke R., Büscher G., Ciotti S., Carbonaro N., Tognetti A. (2016). A multi-modal sensing glove for human manual-interaction studies. Electronics.

[34] Büscher G., Kõiva R., Schürmann C., Haschke R., Ritter H.J. Tactile Dataglove with Fabric-Based Sensors. Proceedings of the 2012 12th IEEE-RAS International Conference on Humanoid Robots (Humanoids 2012).

[35] Michaud H.O., Dejace L., De Mulatier S., Lacour S.P. Design and Functional Evaluation of an Epidermal Strain Sensing System for Hand Tracking. Proceedings of the 2016 IEEE/RSJ International Conference on Intelligent Robots and Systems (IROS).

[36] Chossat J.-B., Tao Y., Duchaine V., Park Y.-L. Wearable soft Artificial Skin for Hand Motion Detection with Embedded Microfluidic Strain Sensing. Proceedings of the 2015 IEEE international conference on robotics and automation (ICRA).

[37] Park W., Ro K., Kim S., Bae J. (2017). A soft sensor-based three-dimensional (3-D) finger motion measurement system. Sensors.

[38] Atalay A., Sanchez V., Atalay O., Vogt D.M., Haufe F., Wood R.J., Walsh C.J. (2017). Batch fabrication of customizable silicone-textile composite capacitive strain sensors for human motion tracking. Adv. Mater. Technol..

[39] Ryu H., Park S., Park J.-J., Bae J. (2018). A knitted glove sensing system with compression strain for finger movements. Smart Mater. Struct..

[40] Glauser O., Panozzo D., Hilliges O., Sorkine-Hornung O. (2019). Deformation capture via soft and stretchable sensor arrays. ACM Trans. Graph..

[41] Glauser O., Wu S., Panozzo D., Hilliges O., Sorkine-Hornung O. (2019). Interactive hand pose estimation using a stretch-sensing soft glove. ACM Trans. Graph..

[42] Yang C.-C., Hsu Y.-L. (2010). A review of accelerometry-based wearable motion detectors for physical activity monitoring. Sensors.

[43] Hsiao P.-C., Yang S.-Y., Lin B.-S., Lee I.-J., Chou W. Data Glove Embedded with 9-axis IMU and Force Sensing Sensors for Evaluation of Hand Function. Proceedings of the 2015 37th annual international conference of the IEEE Engineering in Medicine and Biology Society (EMBC).

[44] O’Flynn B., Sanchez J.T., Connolly J., Condell J., Curran K., Gardiner P., Downes B. Integrated Smart Glove for Hand Motion Monitoring. Proceedings of the Sixth International Conference on Sensor Device Technologies and Applications.

[45] The Humanglove, Humanware. http://www.hmw.it/en/humanglove.html.

[46] Wu J., Huang J., Wang Y., Xing K. (2012). RLSESN-based PID adaptive control for a novel wearable rehabilitation robotic hand driven by PM-TS actuators. Int. J. Intell. Comput. Cybern..

[47] Chen K.-Y., Patel S.N., Keller S. Finexus: Tracking Precise Motions of Multiple Fingertips using Magnetic Sensing. Proceedings of the 2016 CHI Conference on Human Factors in Computing Systems.

[48] The Keyglove. https://github.com/jrowberg/keyglove.

[49] The Hi5 Glove, Noitom. https://hi5vrglove.com/.

[50] Lowe D.G. (2004). Distinctive image features from scale-invariant keypoints. Int. J. Comput. Vis..

[51] Viola P., Jones M. (2001). Rapid object detection using a boosted cascade of simple features. CVPR.

[52] He K., Zhang X., Ren S., Sun J. Deep Residual Learning for Image Recognition. Proceedings of the IEEE Conference on Computer Vision and Pattern Recognition.

[53] Goodfellow I., Pouget-Abadie J., Mirza M., Xu B., Warde-Farley D., Ozair S., Courville A., Bengio Y. Generative Adversarial Nets. Proceedings of the 27th International Conference on Neural Information Processing Systems.

[54] Lu S., Metaxas D., Samaras D., Oliensis J. Using Multiple Cues for Hand Tracking and Model Refinement. Proceedings of the 2003 IEEE Computer Society Conference on Computer Vision and Pattern Recognition.

[55] De La Gorce M., Fleet D.J., Paragios N. (2011). Model-based 3d hand pose estimation from monocular video. IEEE Trans. Pattern Anal. Mach. Intell..

[56] Delamarre Q., Faugeras O. (2001). 3D articulated models and multiview tracking with physical forces. Comput. Vis. Image Underst..

[57] Bray M., Koller-Meier E., Van Gool L. (2007). Smart Particle filtering for high-dimensional tracking. Comput. Vis. Image Underst..

[58] Oikonomidis I., Kyriazis N., Argyros A.A. Efficient Model-Based 3D Tracking of Hand Articulations using Kinect. Proceedings of the 22nd British Machine Vision Conference.

[59] Tkach A., Tagliasacchi A., Remelli E., Pauly M., Fitzgibbon A. (2017). Online generative model personalization for hand tracking. ACM Trans. Graph..

[60] Tagliasacchi A., Schröder M., Tkach A., Bouaziz S., Botsch M., Pauly M. (2015). Robust articulated-ICP for real-time hand tracking. Computer Graphics Forum.

[61] Eberhart R., Kennedy J. Particle Swarm Optimization. Proceedings of the IEEE International Conference on Neural Networks.

[62] Oikonomidis I., Kyriazis N., Argyros A.A. Markerless and Efficient 26-dof Hand Pose Recovery. Proceedings of the Asian Conference on Computer Vision.

[63] Oikonomidis I., Kyriazis N., Argyros A.A. Tracking the Articulated Motion of two Strongly Interacting Hands. Proceedings of the 2012 IEEE Conference on Computer Vision and Pattern Recognition.

[64] Oikonomidis I., Kyriazis N., Argyros A.A. Full DOF Tracking of a Hand Interacting with an Object by Modeling Occlusions and Physical Constraints. Proceedings of the 2011 International Conference on Computer Vision.

[65] Magnenat-Thalmann N., Laperrire R., Thalmann D. Joint-Dependent Local Deformations for Hand Animation and Object Grasping. Proceedings of the Graphics interface’88.

[66] Lewis J.P., Cordner M., Fong N. Pose Space Deformation: A Unified Approach to Shape Interpolation and Skeleton-Driven Deformation. Proceedings of the 27th Annual Conference on Computer Graphics and Interactive Techniques.

[67] Ballan L., Taneja A., Gall J., Van Gool L., Pollefeys M. Motion Capture of Hands in Action using Discriminative Salient Points. Proceedings of the European Conference on Computer Vision.

[68] Sridhar S., Oulasvirta A., Theobalt C. Interactive Markerless Articulated Hand Motion Tracking using RGB and Depth Data. Proceedings of the IEEE International Conference on Computer Vision.

[69] Tzionas D., Srikantha A., Aponte P., Gall J. Capturing Hand Motion with an RGB-D Sensor, Fusing a Generative Model with Salient Points. Proceedings of the 36th German Conference on Pattern Recognition.

[70] Breiman L. (2001). Random forests. Mach. Learn..

[71] Keskin C., Kıraç F., Kara Y.E., Akarun L. Hand Pose Estimation and Hand Shape Classification using Multi-Layered Randomized Decision Forests. Proceedings of the European Conference on Computer Vision.

[72] Tang D., Yu T.-H., Kim T.-K. Real-Time Articulated Hand Pose Estimation using Semi-Supervised Transductive Regression Forests. Proceedings of the IEEE International Conference on Computer Vision.

[73] Liang H., Yuan J., Thalmann D. (2014). Parsing the hand in depth images. IEEE Trans. Multimed..

[74] Tang D., Jin Chang H., Tejani A., Kim T.-K. Latent Regression Forest: Structured Estimation of 3D Articulated Hand Posture. Proceedings of the IEEE Conference on Computer Vision and Pattern Recognition.

[75] Choi M.J., Tan V.Y., Anandkumar A., Willsky A.S. (2011). Learning latent tree graphical models. J. Mach. Learn. Res..

[76] Dollár P., Welinder P., Perona P. Cascaded Pose Regression. Proceedings of the 2010 IEEE Computer Society Conference on Computer Vision and Pattern Recognition.

[77] Sun X., Wei Y., Liang S., Tang X., Sun J. Cascaded Hand Pose Regression. Proceedings of the IEEE Conference on Computer Vision and Pattern Recognition.

[78] Wan C., Yao A., Van Gool L. Hand Pose Estimation from Local Surface Normals. Proceedings of the European Conference on Computer Vision.

[79] Tompson J., Stein M., Lecun Y., Perlin K. (2014). Real-Time Continuous Pose Recovery of Human Hands using Convolutional Networks. ACM Trans. Graph. ToG.

[80] Oberweger M., Wohlhart P., Lepetit V. (2015). Hands Deep in Deep Learning for Hand Pose Estimation. arXiv.

[81] Ge L., Ren Z., Li Y., Xue Z., Wang Y., Cai J., Yuan J. 3D Hand Shape and Pose Estimation from a Single RGB Image. Proceedings of the IEEE Conference on Computer Vision and Pattern Recognition.

[82] Wan C., Probst T., Gool L.V., Yao A. Self-Supervised 3D Hand Pose Estimation through Training by Fitting. Proceedings of the IEEE Conference on Computer Vision and Pattern Recognition.

[83] Baek S., In Kim K., Kim T.-K. Augmented Skeleton Space Transfer for Depth-Based Hand Pose Estimation. Proceedings of the IEEE Conference on Computer Vision and Pattern Recognition.

[84] Oberweger M., Wohlhart P., Lepetit V. (2019). Generalized feedback loop for joint hand-object pose estimation. IEEE Trans. Pattern Anal. Mach. Intell..

[85] Yang L., Yao A. Disentangling Latent Hands for Image Synthesis and Pose Estimation. Proceedings of the IEEE Conference on Computer Vision and Pattern Recognition.

[86] Spurr A., Song J., Park S., Hilliges O. Cross-Modal Deep Variational Hand Pose Estimation. Proceedings of the IEEE Conference on Computer Vision and Pattern Recognition.

[87] Wei S.-E., Ramakrishna V., Kanade T., Sheikh Y. Convolutional Pose Machines. Proceedings of the IEEE Conference on Computer Vision and Pattern Recognition.

[88] Sapp B., Taskar B. Modec: Multimodal Decomposable Models for Human Pose Estimation. Proceedings of the IEEE Conference on Computer Vision and Pattern Recognition.

[89] Johnson S., Everingham M. Learning Effective Human Pose Estimation from Inaccurate Annotation. Proceedings of the CVPR 2011.

[90] Andriluka M., Pishchulin L., Gehler P., Schiele B. 2D Human Pose Estimation: New Benchmark and State of the Art Analysis. Proceedings of the IEEE Conference on Computer Vision and Pattern Recognition.

[91] Cao Z., Simon T., Wei S.-E., Sheikh Y. Realtime Multi-Person 2D Pose Estimation using Part Affinity Fields. Proceedings of the IEEE Conference on Computer Vision and Pattern Recognition.

[92] Henia O.B., Hariti M., Bouakaz S. A Two-Step Minimization Algorithm for Model-Based Hand Tracking. Proceedings of the 18th International Conference on Computer Graphics, Visualization and Computer Vision (WSCG).

[93] Girshick R. Fast R-CNN. Proceedings of the IEEE International Conference on Computer Vision.

[94] Misra I., Shrivastava A., Gupta A., Hebert M. Cross-Stitch Networks for Multi-Task Learning. Proceedings of the IEEE Conference on Computer Vision and Pattern Recognition.

[95] Zhang Z., Luo P., Loy C.C., Tang X. Facial Landmark Detection by Deep Multi-Task Learning. Proceedings of the European Conference on Computer Vision.

[96] Simon T., Joo H., Matthews I., Sheikh Y. Hand Keypoint Detection in Single Images using Multiview Bootstrapping. Proceedings of the IEEE Conference on Computer Vision and Pattern Recognition.

[97] Joo H., Liu H., Tan L., Gui L., Nabbe B., Matthews I., Kanade T., Nobuhara S., Sheikh Y. Panoptic Studio: A Massively Multiview System for Social Motion Capture. Proceedings of the IEEE International Conference on Computer Vision.

[98] Zimmermann C., Brox T. Learning to Estimate 3D Hand Pose from Single RGB Images. Proceedings of the IEEE International Conference on Computer Vision.

[99] Iqbal U., Molchanov P., Breuel Juergen Gall T., Kautz J. Hand Pose Estimation via Latent 2.5 d Heatmap Regression. Proceedings of the European Conference on Computer Vision (ECCV).

[100] Rad M., Oberweger M., Lepetit V. Domain Transfer for 3d Pose Estimation from Color Images without Manual Annotations. Proceedings of the 14th Asian Conference on Computer Vision.

[101] Cai Y., Ge L., Cai J., Yuan J. Weakly-Supervised 3D Hand Pose Estimation from Monocular RGB Images. Proceedings of the European Conference on Computer Vision (ECCV).

[102] Ge L., Liang H., Yuan J., Thalmann D. (2018). Robust 3D hand pose estimation from single depth images using multi-view CNNs. IEEE Trans. Image Process..

[103] Wang P.-S., Liu Y., Guo Y.-X., Sun C.-Y., Tong X. (2017). O-CNN: Octree-based convolutional neural networks for 3d shape analysis. ACM Trans. Graph..

[104] Che Y., Song Y., Qi Y. A Novel Framework of Hand Localization and Hand Pose Estimation. Proceedings of the ICASSP 2019—2019 IEEE International Conference on Acoustics, Speech and Signal Processing (ICASSP).

[105] Ge L., Ren Z., Yuan J. Point-to-Point Regression Pointnet for 3D Hand Pose Estimation. Proceedings of the European Conference on Computer Vision (ECCV).

[106] Ge L., Cai Y., Weng J., Yuan J. Hand PointNet: 3D Hand Pose Estimation using Point Sets. Proceedings of the IEEE Conference on Computer Vision and Pattern Recognition.

[107] Dou Y., Wang X., Zhu Y., Deng X., Ma C., Chang L., Wang H. Cascaded Point Network for 3D Hand Pose Estimation. Proceedings of the ICASSP 2019—2019 IEEE International Conference on Acoustics, Speech and Signal Processing (ICASSP).

[108] Li S., Lee D. Point-to-Pose Voting Based Hand Pose Estimation using Residual Permutation Equivariant Layer. Proceedings of the IEEE Conference on Computer Vision and Pattern Recognition.

[109] Deng X., Yang S., Zhang Y., Tan P., Chang L., Wang H. (2017). Hand3D: Hand pose estimation using 3d neural network. arXiv.

[110] Ge L., Liang H., Yuan J., Thalmann D. 3D Convolutional Neural Networks for Efficient and Robust Hand Pose Estimation from Single Depth Images. Proceedings of the IEEE Conference on Computer Vision and Pattern Recognition.

[111] Moon G., Chang J.Y., Lee K.M. V2v-Posenet: Voxel-to-Voxel Prediction Network for Accurate 3D Hand and Human Pose Estimation from a Single Depth Map. Proceedings of the IEEE Conference on Computer Vision and Pattern Recognition.

[112] Ge L., Liang H., Yuan J., Thalmann D. (2018). Real-time 3D hand pose estimation with 3D convolutional neural networks. IEEE Trans. Pattern Anal. Mach. Intell..

[113] Yuan S., Ye Q., Garcia-Hernando G., Kim T.-K. (2017). The 2017 hands in the million challenge on 3d hand pose estimation. arXiv.

[114] Haque A., Peng B., Luo Z., Alahi A., Yeung S., Fei-Fei L. Towards Viewpoint Invariant 3d Human Pose Estimation. Proceedings of the European Conference on Computer Vision.

[115] Zhang J., Jiao J., Chen M., Qu L., Xu X., Yang Q. (2016). 3D hand pose tracking and estimation using stereo matching. arXiv.

[116] Sridhar S., Mueller F., Zollhöfer M., Casas D., Oulasvirta A., Theobalt C. Real-Time Joint Tracking of a Hand Manipulating an Object From RGB-D Input. Proceedings of the European Conference on Computer Vision.

[117] Mueller F., Mehta D., Sotnychenko O., Sridhar S., Casas D., Theobalt C. Real-Time Hand Tracking under Occlusion from an Egocentric RGB-D Sensor. Proceedings of the IEEE International Conference on Computer Vision.

[118] Hinterstoisser S., Lepetit V., Ilic S., Holzer S., Bradski G., Konolige K., Navab N. Model Based Training, Detection and Pose Estimation of Texture-Less 3d Objects in Heavily Cluttered Scenes. Proceedings of the Asian Conference on Computer Vision.

[119] Baek S., Kim K.I., Kim T.-K. Pushing the Envelope for RGB-Based Dense 3D Hand Pose Estimation via Neural Rendering. Proceedings of the IEEE Conference on Computer Vision and Pattern Recognition.

[120] Romero J., Tzionas D., Black M. (2017). Embodied hands: Modeling and capturing hands and bodies together. ACM Trans. Graph..

[121] Mueller F., Davis M., Bernard F., Sotnychenko O., Verschoor M., Otaduy M.A., Casas D., Theobalt C. (2019). Real-time pose and shape reconstruction of two interacting hands with a single depth camera. ACM Trans. Graph..

[122] Boukhayma A., Bem R.D., Torr P.H. 3D Hand Shape and Pose from Images in the Wild. Proceedings of the IEEE Conference on Computer Vision and Pattern Recognition.

[123] Zhang H., Bo Z.-H., Yong J.-H., Xu F. (2019). Interaction fusion: Real-time reconstruction of hand poses and deformable objects in hand-object interactions. ACM Trans. Graph..

[124] Yuan S., Ye Q., Stenger B., Jain S., Kim T.-K. Bighand2. 2m Benchmark: Hand Pose Dataset and State of the Art Analysis. Proceedings of the IEEE Conference on Computer Vision and Pattern Recognition.

[125] Wetzler A., Slossberg R., Kimmel R. (2015). Rule of thumb: Deep derotation for improved fingertip detection. arXiv.

[126] Sharp T., Keskin C., Robertson D., Taylor J., Shotton J., Kim D., Rhemann C., Leichter I., Vinnikov A., Wei Y. Accurate, Robust, and Flexible Real-Time Hand Tracking. Proceedings of the 33rd Annual ACM Conference on Human Factors in Computing Systems.

[127] Tekin B., Bogo F., Pollefeys M. H+ O: Unified Egocentric Recognition of 3D Hand-Object Poses and Interactions. Proceedings of the IEEE Conference on Computer Vision and Pattern Recognition.

[128] Myanganbayar B., Mata C., Dekel G., Katz B., Ben-Yosef G., Barbu A. Partially Occluded Hands: A Challenging New Dataset for Single-Image Hand Pose Estimation. Proceedings of the 14th Asian Conference on Computer Vision (ACCV 2018).

